# Case report: Clinical characteristics of two cases of pneumonia caused with different strains of *Chlamydia psittaci*

**DOI:** 10.3389/fcimb.2023.1086454

**Published:** 2023-01-31

**Authors:** Zheng Wang, Chen Chen, Hu Lu, Li Wang, Lin Gao, Jing Zhang, Chi Zhu, Furong Du, Lunbiao Cui, Yan Tan

**Affiliations:** ^1^ Department of Respiratory and Critical Care Medicine, Nanjing First Hospital, Nanjing Medical University, Nanjing, China; ^2^ Department of Respiratory and Critical Care Medicine, The Second Affiliated Hospital of Soochow University, Suzhou, China; ^3^ Department of Emergency, The Second Hospital of Nanjing, Nanjing University of Chinese Medicine, Nanjing, China; ^4^ State Key Laboratory of Translational Medicine and Innovative Drug Development, Jiangsu Simcere Diagnostics Co., Ltd., Nanjing, China; ^5^ NHC Key Laboratory of Enteric Pathogenic Microbiology, Jiangsu Provincial Center for Disease Control and Prevention, Nanjing, China

**Keywords:** *Chlamydia psittaci*, genotype, clinical characteristics, case report, zoonosis

## Abstract

**Background:**

With the development of metagenomic sequencing technologies, more and more cases of pneumonia caused with *Chlamydia psittaci* (*C. psittaci*) have been reported. However, it remains unknown about the characteristics of patients with pneumonia caused by different strains of *C. psittaci*. Here, we shared the clinical characteristics of two cases of pneumonia caused with *C. psittaci* strains SZ18-2 and SZ15 which were rarely identified in humans.

**Case presentation:**

Case 1: A 69-year-old male farmer who fed ducks presented to hospital for cough, diarrhea and lethargy with the temperature of 39.8°C. Case 2: A 48-year-old male worker who slaughtered ducks was transferred to hospital for high fever, cough, myalgia, diarrhea and loss of appetite. Both patients did not take any protective measures (wearing face masks or gloves) while processing ducks. *C. psittaci* pneumonia was diagnosed by metagenomic next-generation sequencing and polymerase chain reaction. After treatment with doxycycline and azithromycin individually, they recovered well and discharged from hospital. Through OmpA sequencing, two different strains of SZ18-2 and SZ15 were identified in case 1 and case 2, respectively.

**Conclusions:**

Patients infected with different strains of *C. psittaci* may own different clinical manifestations. *C. psittaci* infection should be suspected when pneumonia appears, accompanied by digestive symptoms and multiple organ dysfunction, especially under the exposure of specific birds.

## Background

Psittacosis is a zoonotic infectious disease resulting from the transmission of the bacterium *Chlamydia psittaci* (*C. psittaci*) from birds to humans. Humans infected with psittacosis may experience non-specific flu-like symptoms by directly contacting with infected animals or by inhaling their excreta, such as high fever, cough, myalgia, fatigue, and loss of appetite (Knittler and Sachse, 2015; Chen et al., 2020). Human psittacosis is often overlooked by clinicians due to atypical clinical manifestations, low incidence, and limited testing methods. According to a meta-analysis in 2017 (Hogerwerf et al., 2017), approximately 1% of annual community-acquired pneumonia was caused by *C. psittaci* worldwide. At present, however, there are lack of assays for identification of *C. psittaci* pathogens in various laboratories, thus it is very difficult and time-consuming for the diagnosis of *C. psittaci* infection.

*C. psittaci* is commonly classified into 15 genotypes, including A, B, C, D, E, F, E/B, M56, 1V, WC, 6N, Matl16, R54, YP84 and CPX0308 (Radomski et al., 2016), which are usually identified through the outer membrane protein A (ompA) sequencing. *C. psittaci* genotypes have some preference for specific species (Zaręba-Marchewka et al., 2021). In recent years, more and more cases of *C. psittaci* pneumonia have been reported with the development of metagenomic sequencing technologies. However, it remains unknown regarding the characteristics of human pneumonia caused by different strains of *C. psittaci*. Here, we described the clinical characteristics of two patients with pneumonia caused by different strains of *C. psittaci* (SZ18-2 and SZ15), which had been previously detected in ducks but rarely reported in human (Beeckman and Vanrompay, 2009; Lin et al., 2019).

## Case presentation

**Figure 1:**
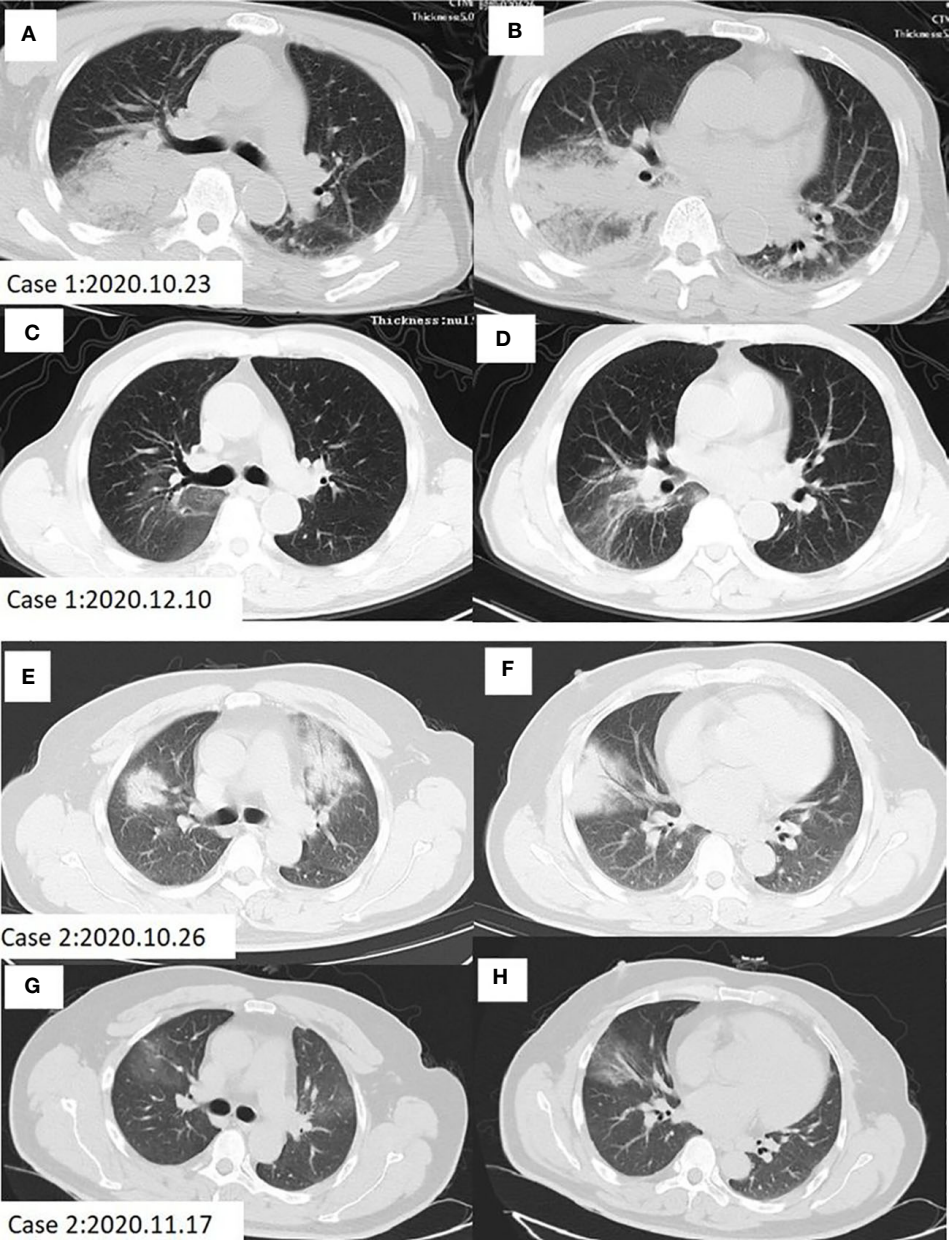
Chest CT scan of Case 1 and Case 2 before and after treatment. **(A, B)** Patchy infiltration and consolidation of both lungs, especially in the right lung in case 1 before treatment. **(C, D)** Lung shadow absorption after treatment in case 1. **(E, F)** Patchy high-density shade was seen in the anterior segment of the upper lobe, median lobe and the basal segment of the inferior lobe in the right lung, as well as the anterior segment of the upper lobe in the left lung of case 2 before treatment. **(G, H)** Lung shadow absorption after treatment in case 2.

**Case 1. A** 69-year-old man came to our hospital on October 23, 2020 for cough and asthma for 8 days, inflammatory infiltration in multiple lobes, and disturbance of consciousness. He was diagnosed as severe pneumonia, accompanied by septic shock and pleural effusion. Eight days ago, the patient developed cough with sticky sputum, myalgia, loss of appetite and diarrhea for 4-5 times per day, but the symptoms didn’t improve after intravenous transfusion of cefazoxime. On the next day, he began to experience dyspnea and lethargy, with the highest temperature of 39.8°C. He had no history of smoking, alcohol consumption or drug use. Moist rales can be heard in the right lung. Laboratory examinations showed elevated levels of creatine kinase (CK), lactic dehydrogenase (LDH), alanine aminotransferase (ALT), aspartate aminotransferase (AST), creatinine (Cr) and blood urea nitrogen (BUN). The chest computed tomography (CT) revealed patchy consolidation in both lungs, especially in the right lung (**Figures 1A, B**).

**Case 2. A** 48-year-old man with a long-term history of hypertension presented to our hospital on October 26, 2020 due to high fever, cough, muscle pain in the whole body, diarrhea and loss of appetite for 5 days, and was diagnosed as severe pneumonia. This patient was also treated with intravenous transfusion of cefazoxime, but the symptoms did not improve. No moist rales were heard by the lung auscultation. Laboratory examinations showed elevated levels of CK, LDH, ALT and AST on admission. The chest CT indicated a patchy high-density shade in the anterior segment of the upper lobe, median lobe, and the basal segment of the inferior lobe in the right lung, as well as the anterior segment of the upper lobe in the left lung (**Figures 1E, F**).

Notably, these two patients both had a history of duck contact, and did not take any protective measures, such as wearing face masks or gloves, while processing the ducks. The clinical features and laboratory indicators of the two patients on admission are summarized in **Table 1**.

**Table 1:** Clinical features and laboratory indicators of two patients with *C. psittaci* pneumonia on admission.

Characteristics	Case 1	Case 2
Demographics		
Gender	Male	Male
Age (years)	69	48
Clinical manifestations		
Highest fever (°C)	39.8	40
Respiratory rate, times/min	30	30
Blood pressure (mmHg)	137/80	144/77
Pulse rate, times/min	70	102
Laboratory tests		
Oxygenation index	448	320
WBC (10^9^/L)	5.94	9.96
Percentage of neutrophils	93	94
Hs-CRP (mg/L)	0.5	195.15
CK (ng/mL)	2141	300.6
LDH (U/L)	1476	507
ALT (U/L)	137	92
AST (U/L)	334	177.8
Cr (umol/L)	210.8	99
BUN (mmol/L)	21.54	6.69

**Figure 2:**
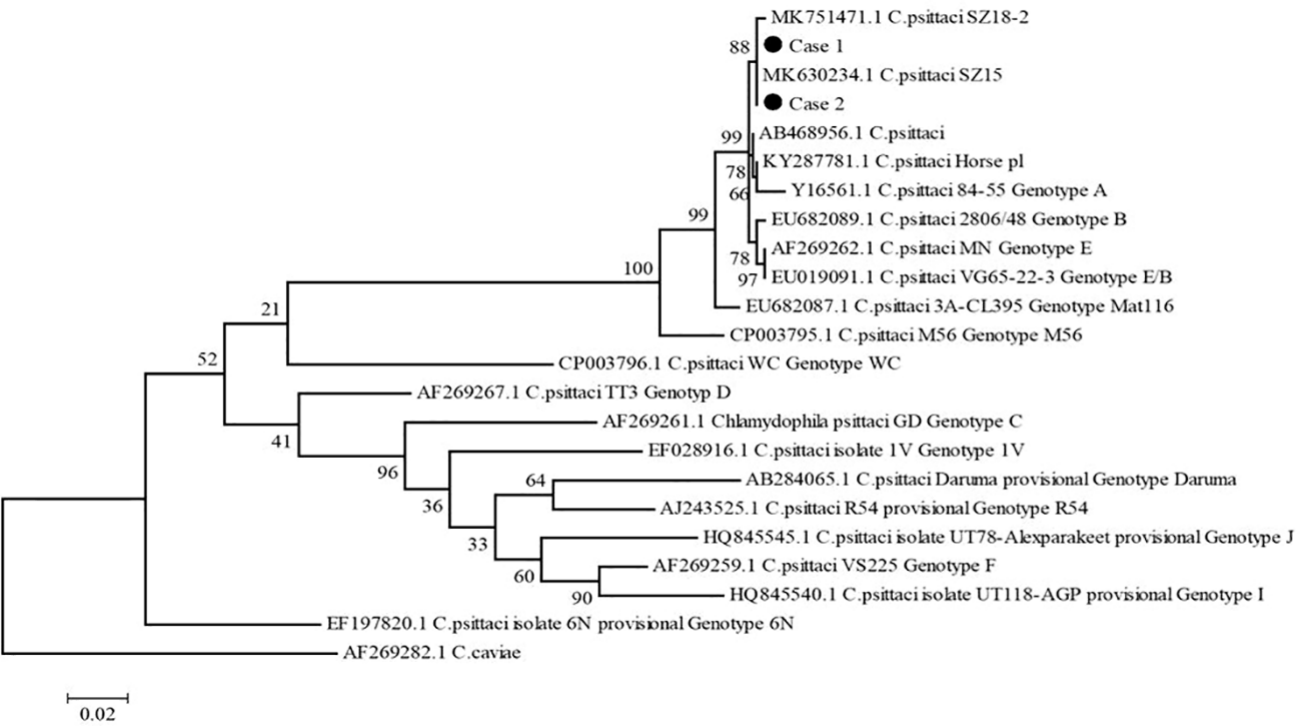
Phylogenetic tree of *C. psittaci* ompA gene sequences. The tree was constructed using the maximum-likelihood method based on GTR model, with 1,000 bootstrap replicates in MEGA5, compared with reference sequences available in the GenBank database (GenBank accession number indicated). C. caviae (AF269282.1) was used as an out-group. Black dots indicate isolates from this study. Numbers at nodes indicate bootstrap values. Scale bar indicates nucleotide substitutions per site.

To explore the possible pathogens of the infection, we obtained the bronchoalveolar lavage fluid (BALF) from two patients by electronic bronchoscope on the 3^rd^ day after admission. The BALF was delivered for culture and metagenomic next-generation sequencing (mNGS). The results of culture were negative, but *C. psittaci* was identified by mNGS, which was further confirmed by real-time polymerase chain reaction (RT-PCR) assay. PCR primer sequence was F: ATGAAAAAACTCTTGAAATCGG and R: CAAGATTTTCTAGA CTTCATTTT. To determine the genotypes of *C. psittaci* from 2 cases, 1100 bp fragments of the omp A gene were amplified using the nest PCR (Herrmann et al., 2006) and sequenced using an Applied Biosystems 3130 Genetic Analyzer (ThermoFisher Scientific, https://www.thermofisher.com). We aligned nucleotide sequences using Mafft (https://mafft.cbrc.jp/alignment/software/) and constructed a phylogenetic tree with MEGA5 (https://www.megasoftware.net) according to a previous study (Lu et al., 2019). The results showed 100% identical omp A gene sequences in these two cases, with 100% and 99.81% identical to the sequence of *C. psittaci* strain SZ15 (GenBank accession no. MK630234.1) and SZ18-2 (GenBank accession no. MK751471.1), respectively (**Figure 2**). The processes of mNGS and RT-PCR are described particularly in **Supplementary Materials**.

**Figure 3:**
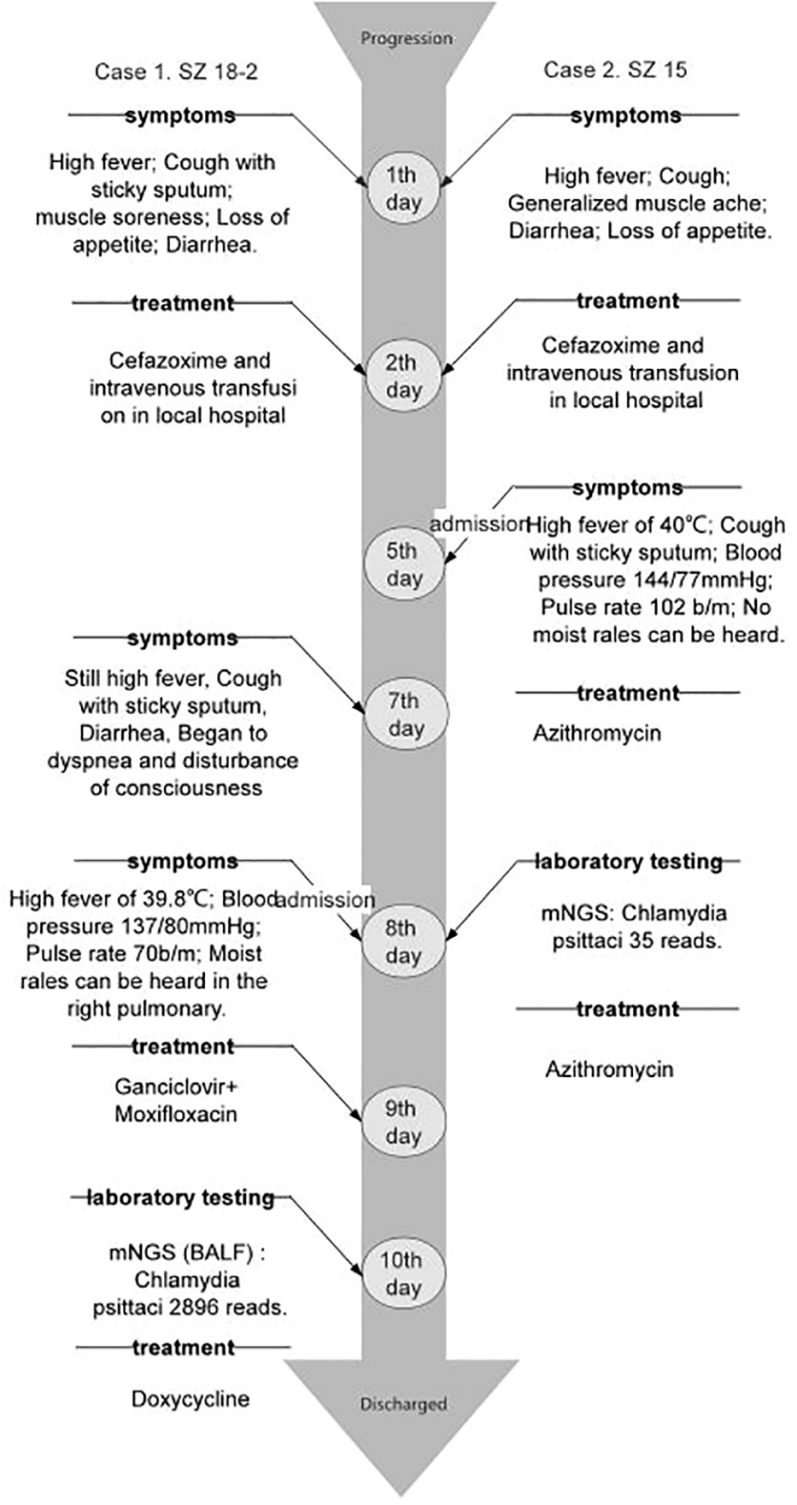
The timeline of disease progression and treatment of the two patients.

Two cases of pneumonia were both confirmed to be caused by *C. psittaci*. Case 1 was treated with oral administration of doxycycline, while case 2 was treated with intravenous injection of azithromycin. The clinical symptoms and laboratory indicators were both improved after treatment. The CT also showed decreased inflammatory exudation after 1-2 months of treatment (**Figures 1C, D, G, H**). During the treatment, there were no adverse and unanticipated events. The timelines of disease progression and treatment are depicted in **Figure 3**, and the changes of laboratory indicators are described in **Figure S1**.

## Discussion

*C. psittaci* is a zoonotic pathogen which can infect avian and mammalian hosts, including humans. In clinical practice, however, *C. psittaci* infection is very easy to be overlooked because of the difficulty in diagnosis. In this study, we reported the clinical characteristics and laboratory indicators of two patients with pneumonia caused by different strains of *C. psittaci*, and demonstrated that patients infected with different strains of *C. psittaci* may own different clinical manifestations, which will need more large-scale studies to further verify.

Patients with *C. psittaci* infection may experience multiple organ dysfunction, for which several hypotheses have been proposed. It is reported that the nuclear factor-κB signaling pathway is activated and the function of macrophages is inhibited at the time of *C. psittaci* infection (Chu et al., 2020), and the microRNA expression of inflammatory cytokines in human bronchial epithelial cells is upregulated (Liu et al., 2021). In 2016, Chu et al. found that virulent *C. psittaci* infection could inhibit the immune response through suppression of humoral responses and adjustment of Th1/Th2 balance, consequently leading to increased mortality in H9N2-infected birds (Chu et al., 2016).

In this study, we reported two patients infected with different strains of *C. psittaci* (SZ18-2 and SZ15). The SZ15 isolate is closely related to the VS1, 6BC and 84‐55 strains, indicating that the SZ15 isolate belongs to the genotype A, which is usually found in psittacine birds (Lin et al., 2019). Based on Genbank phylogenetic relationship of *C. psittaci* strains, SZ15 and SZ 18-2 were clustered together. Further analysis is needed to determine whether these two strains belong to the same genotype.

In this study, the two patients suffered from soreness and weakness in the whole body and more pronounced digestive symptoms rather than respiratory symptoms. The white blood cells were in normal range, while the percentage of neutrophils increased consistently in both cases. The high-sensitivity C-reactive protein levels were normal in case 1 on day 9 after symptoms occurred, but significantly elevated on day 14 and were markedly higher than that in case 2 on day 5. In addition, they both had an abnormal rise in liver enzyme and myocardial enzyme, suggesting that the pulmonary infection caused by *C. psittaci* might damage the liver and heart function to some extent. The concentration of Cr and BUN only increased in case 1, which may be related to the lethargy in case 1. Moist rales were found in case 1 but not in case 2.

*C. psittaci* infection should be suspected when pneumonia was present, accompanied by digestive manifestations and multiple organ dysfunction, especially under the exposure of specific birds. By means of mNGS, we found the main pathogen and took therapeutic measures in time. Both patients recovered finally. These two cases in our study had close contact with ducks before the onset, suggesting that *C. psittaci* pneumonia might be associated to prior exposure to ducks. However, the duck samples were not collected and detected to confirm the source of *C. psittaci*. In the future, this association will need to be further investigated.

In 2019, Lin et al. isolated *C. psittaci* strain SZ15 (genotype A) in ducks (Lin et al., 2019). The ducks inoculated with SZ15 showed anorexia, depression, and poor laying performance. Besides, hemorrhage in the liver, heart, oviduct, and ovary was also observed. The infected humans and ducks shared some similarity in manifestations. This reminds us to enhance the surveillance of *C. psittaci* in ducks and proper respiratory protection should be taken for those feeding or slaughtering ducks.

In conclusion, patients with pneumonia caused by different strains of *C. psittaci* may own different clinical manifestations. *C. psittaci* infection should be suspected when pneumonia appears, accompanied by digestive symptoms and multiple organ dysfunction, especially under the exposure of specific birds.

## Data availability statement

The datasets presented in this study can be found in online repositories. The names of the repository/repositories and accession number(s) can be found in the article/**Supplementary Material**.

## Ethics statement

This study involving human participants was reviewed and approved by the Ethics Committee of Nanjing First Hospital (NO. KF20220516-05).

## Author contributions

LW, LC, and CC Wrote the first draft of the manuscript and critically reviewed and revised the manuscript. LG, HL, and ZW provided these cases. YT have revised it and approved the submitted version. LG, JZ, CZ, and FD participate in the discussion. YT and LC designed and provided the funding to this research separately. All authors contributed to the article and approved the submitted version.

## References

[B1] Beeckman D. S. Vanrompay D. C. (2009). Zoonotic chlamydophila psittaci infections from a clinical perspective. Clin. Microbiol. Infect. 15 (1), 11–17. doi: 10.1111/j.1469-0691.2008.02669.x 19220335

[B2] Chen X. Cao K. Wei Y. Qian Y. Liang J. Dong D. . (2020). Metagenomic next-generation sequencing in the diagnosis of severe pneumonias caused by chlamydia psittaci. Infection 48 (4), 535–542. doi: 10.1007/s15010-020-01429-0 32314307 PMC7223968

[B3] Chu J. Zhang Q. Zhang T. Han E. Zhao P. Khan A. . (2016). Chlamydia psittaci infection increases mortality of avian influenza virus H9N2 by suppressing host immune response. Sci. Rep. 6, 29421. doi: 10.1038/srep29421 27405059 PMC4941526

[B4] Chu J. Li X. Qu G. Wang Y. Li Q. Guo Y. . (2020). *Chlamydia psittaci* PmpD-n exacerbated chicken macrophage function by triggering Th2 polarization and the TLR2/MyD88/NF-κB signaling pathway. Int. J. Mol. Sci. 21 (6), 2003. doi: 10.3390/ijms21062003 32183481 PMC7139469

[B5] Herrmann B. Persson H. Jensen J. K. Joensen H. D. Klint M. Olsen B. (2006). Chlamydophila psittaci in fulmars, the faroe islands. Emerg. Infect. Dis. 12 (2), 330–332. doi: 10.3201/eid1202.050404 16494766 PMC3373105

[B6] Hogerwerf L. De Gier B. Baan B. Van Der Hoek W. (2017). Chlamydia psittaci (psittacosis) as a cause of community-acquired pneumonia: a systematic review and meta-analysis. Epidemiol. Infect. 145 (15), 3096–3105. doi: 10.1017/S0950268817002060 28946931 PMC9148753

[B7] Knittler M. R. Sachse K. (2015). Chlamydia psittaci: update on an underestimated zoonotic agent. Pathog. Dis. 73 (1), 1–15. doi: 10.1093/femspd/ftu007 25853998

[B8] Lin W. Chen T. Liao L. Wang Z. Xiao J. Lu J. . (2019). A parrot-type chlamydia psittaci strain is in association with egg production drop in laying ducks. Transbound Emerg. Dis. 66 (5), 2002–2010. doi: 10.1111/tbed.13248 31127977

[B9] Liu L. Chen X. Tang T. Chen L. Huang Q. Li Z. . (2021). Analysis of microRNA expression profiles in human bronchial epithelial cells infected by chlamydia psittaci. Microb. Pathog. 154, 104837. doi: 10.1016/j.micpath.2021.104837 33689813

[B10] Lu B. Cui L. B. Gu M. H. Shi C. Sun C. W. Zhao K. C. . (2019). Outbreak of vaccinia virus infection from occupational exposure, China, 2017. Emerg. Infect. Dis. 25 (6), 1192–1195. doi: 10.3201/eid2506.171306 31107220 PMC6537725

[B11] Radomski N. Einenkel R. Müller A. Knittler M. R. (2016). Chlamydia-host cell interaction not only from a bird's eye view: some lessons from chlamydia psittaci. FEBS Lett. 590 (21), 3920–3940. doi: 10.1002/1873-3468.12295 27397851

[B12] Zaręba-Marchewka K. Szymańska-Czerwińska M. Livingstone M. Longbottom D. Nieczuk K. (2021). Whole genome sequencing and comparative genome analyses of *Chlamydia abortus* strains of avian origin suggests that *Chlamydia abortus* species should be expanded to include avian and mammalian subgroups. Pathogens 10 (11), 1405. doi: 10.3390/pathogens10111405 34832561 PMC8623937

