# Supplementary material for: Case report: Clinical characteristics of two cases of pneumonia caused with different strains of Chlamydia psittaci
Source: Front Cell Infect Microbiol. 2023 Jan 31;13:1086454. doi: 10.3389/fcimb.2023.1086454 (PMC9927004; doi:10.3389/fcimb.2023.1086454)
Supplement: Supplementary file 1 [file DataSheet_1.docx]

**[Supplementary material](javascript:;)s**

**mNGS**

**DNA extraction:** First, transfer 1-2 mL of BALF sample to a clean centrifuge tube of 2 mL and centrifuge at 14 000 g for 5 min. Second, carefully aspirate the supernatant, and keep 200 µL of the supernatant and pellet in a centrifuge tube for later use. Third, add 10 µL of the prepared lysozyme (20 mg/mL) and incubate at 37°C for 15 min, then put in a MP Lysing Matri E tube and add 200 µL of GB lysis solution. Next, shake the MP tube on a wall breaker (FastPrep-24™ 5G) at 6 m/s for 120 s, and centrifuge at 14 000 g at low temperature for 5 min. After centrifugation, take all the supernatant and add it to a new EP tube of 2 mL. Finally, use a micro-sample genomic DNA extraction kit (DP316, Tiangen) to extract the nucleic acid.

**Library preparation and sequencing:** NEBNext Ultra II DNA Library Prep Kit (New England Biolabs Inc.) was used to construct Illumina sequencing libraries and Nextseq 550 DX (75 bp single-end reads; Illumina) was used for sequencing. About 20 million reads were generated for each sample. About 0.8 G of data were generated for each sample.

**Bioinformatics analysis:** High-quality sequencing data were generated by removing low-quality reads, including adapter contamination, duplicated reads and short reads (Illumina: length < 50 bp). An alignment tool (Burrows-Wheeler Alignment) was used to map to a human reference genome (GRCh38) to exclude human sequence data. The remaining sequencing data were aligned to NCBI nt database by SNAP. The mapped data were processed for advanced data analysis with in-house scripts, including taxonomy annotation, genome coverage/depth calculation and abundance calculation.

| **Reagents** | [**Volume**](javascript:;) | [**Final concentration**](javascript:;) |
| --- | --- | --- |
| 2X One Step RT-PCR Buffer Ⅲ | 10 μL |  |
| TaKaRa Ex Taq HS (5 U/μL) | 0.4 μL |  |
| PrimeScript RT Enzyme Mix Ⅱ | 0.4 μL |  |
| PCR Forward Primer (10 μM) | 0.4 μL | 0.2 μM |
| PCR Reverse Primer (10 μM) | 0.4 μL | 0.2 μM |
| Probe | 0.4 μL | 0.2-0.4 μM |
| Total RNA | 2 μL |  |
| RNase Free dH2O | 6 μL |  |
| Total | 20 μL |  |

**RT-PCR process**

The PCR amplification procedure was a total of 46 cycles with 42℃ for 5 min, 95℃ for 10 s, 95℃ for 5 s and 60℃ for 34 s. FAM fluorescence signals were collected at the end of each cycle.

**Figure S1**. Changes of laboratory indicators during disease progression
